# Impacts of Continuous Cropping on Fungal Communities in the Rhizosphere Soil of Tibetan Barley

**DOI:** 10.3389/fmicb.2022.755720

**Published:** 2022-02-04

**Authors:** Youhua Yao, Yuan Zhao, Xiaohua Yao, Yixiong Bai, Likun An, Xin Li, Kunlun Wu

**Affiliations:** ^1^Academy of Agricultural and Forestry Sciences, Qinghai University, Xining, China; ^2^Qinghai Key Laboratory of Hulless Barley Genetics and Breeding, Xining, China; ^3^Qinghai Subcenter of National Hulless Barley Improvement, Xining, China; ^4^College of Eco-Environmental Engineering, Qinghai University, Xining, China

**Keywords:** Tibetan barley, continuous cropping, rhizosphere soil, fungal community structure, co-occurrence network

## Abstract

Microbial community structures and keystone species play critical roles in soil ecological processes; however, their responses to the continuous cropping of plants are virtually unknown. Here, we investigated the community dynamics and keystone species of fungal communities in the rhizosphere soils of continuously cropped Tibetan barley (a principal cereal cultivated on the Qinghai–Tibetan Plateau). We found that the Chao1 and Phylogenetic Diversity (PD) indices decreased with increased cropping years. The relative abundance of the genera *Cystofilobasidium*, *Mucor*, and *Ustilago* increased with the extension of continuous cropping years, whereas *Fusarium* showed the opposite pattern. Furthermore, long-term monocropped Tibetan barley simplified the complexity of the co-occurrence networks. Keystone operational taxonomic units (OTUs) changed with continuous cropping, and most of the keystone OTUs belonged to the phylum *Ascomycota*, suggesting their important roles in rhizosphere soil. Overall, this study revealed that the continuous cropping of Tibetan barley impacted both on the richness, phylogenetic diversity, and co-occurrence network of fungal community in the rhizosphere. These findings enhance our understanding of how rhizosphere fungal communities respond to monocropped Tibetan barley.

## Introduction

Continuous cropping is the agricultural practice of the long-term cultivation of an identical crop in the same soil. This is an unsustainable agriculture practice that results in soil degeneration and reduced crop yield and quality (Pervaiz et al., 2020). Although the advantages of crop rotation and inter-cropping in improving soil nutrients and enhancing crop yields are well known, continuous cropping is still common in modern agricultural systems due to several factors, such as limited arable land, economic interests, and climatic constraints (Pervaiz et al., 2020). Tibetan barley (*Hordeum vulgare* L., known as “*qingke*” in China) serves as a staple food and important livestock feed on the Tibetan Plateau (Guedes et al., 2015). It is drought- and cold-tolerant, exhibiting strong adaptability to the extreme climate of the plateau (Zeng et al., 2018). The cropping area of Tibetan barely accounts for approximately 43% of the grain crop area on the Tibetan Plateau (Zhao et al., 2015). Owing to the increasing demand for barley production and the shrinking cultivatable area, the continuous cropping of Tibetan barley is a very common agricultural practice on the Tibetan Plateau (Zeng et al., 2018).

Rhizosphere soil microorganisms affect the growth, development, and health of plants (Mohanram and Kumar, 2019). Among these soil organisms, rhizosphere fungi are important decomposers, having important roles in balancing the rhizosphere ecosystem (Qin et al., 2017). It has been shown that continuous cropping alters the fungal community structure of the rhizosphere soil of sweet potato (Gao et al., 2019), tobacco (Wang et al., 2020), and sugarcane (Pang et al., 2021). However, these crops are usually cultivated in normal-altitude regions, and the role of continuous cropping in shaping the microbiome structure of plants cultivated at high altitudes remains poorly investigated.

Previous studies have focused on the effects of continuous cropping on microbial community composition and diversity, as well as their responses to soil properties, plant species, or plant genotypes (He et al., 2016; Zhang et al., 2016a; Pervaiz et al., 2020). However, microbial communities encompass not only the number of species and their abundances under different environmental conditions but also the complex ecological interactions among different species (Zhou et al., 2011). Co-occurrence network analysis offers new insight into the inter-species interactions of soil microbial communities and promotes the understanding of the niche spaces among community members (Barberán et al., 2012). Previous studies have proved that continuous cropping disturbed the fungal community structure in the rhizosphere soil, and loss of biodiversity has been identified as a common phenomenon in soils with long-term continuous cropping (Pervaiz et al., 2020). Yet, less attention has been paid to the co-occurrence networks and keystone species among fungal communities resulting from the continuous cropping of plants cultivated in high-altitude regions, such as Tibetan barley.

Understanding the influence of continuous cropping on the fungal community in the soil rhizosphere is pivotal for designing effective farm management practices to relieve the soil degradation associated with Tibetan barley cultivation. Therefore, we hypothesized that the long-term continuous cropping of Tibetan barley alters the fungal community structure in rhizosphere soils, resulting in specific responses of rhizosphere soil fungi to continuous cropping. In this study, we evaluated fungal communities in cultivated Tibetan barley fields with 2–6 years of monoculture history. The objectives of our study were to explore the dynamics of rhizosphere fungal diversity and composition during the continuous cropping of Tibetan barley. Further, we evaluated the changes in co-occurrence patterns and keystone species throughout continuous cropping. Our study should provide a better understanding of the response and interaction of the rhizosphere fungal community to the continuous cropping of plants cultivated in high-altitude regions.

## Materials and Methods

### Study Site Description and Soil Sampling

The Tibetan barley field was located at the Quankou Experimental Station, Menyuan Hui Autonomous County, Qinghai Province, China (37°21′N, 101°44′E). Meteorological data, which were obtained from National Meteorological Observatory (NOW) stations in Qinghai, included a comprehensive dataset for monthly values for observed precipitation and mean air temperature between 2011 and 2016. The experimental field has mean annual precipitation of 530–560 mm, an average annual sunshine duration of 2,575 h, an average annual temperature of 1.3°C, and annual evaporation of 1,128–1,343 mm. The soil in the field is a Kastanozem according to the FAO classification (Food Agriculture Organization of the United Nations, 1990), with 4.5 g kg^−1^ total nitrogen, 1.0 g kg^−1^ total phosphorus, 22.0 g kg^−1^ total potassium, 60.5 g kg^−1^ organic matter, 245 mg kg^−1^ available nitrogen, 20 mg kg^−1^ available phosphorus, and 200 mg kg^−1^ available potassium. The experimental field contained four plots, each 10.0 m × 10.0 m in size. From 2011 to 2016, Tibetan barley (*Hordeum vulgare* L., *qingke*) was planted every year in March and harvested in September. Blended fertilizer with 22.5 g m^−2^ (NH_4_)_2_SO_4_ and 11.5 g m^−2^ urea was applied annually before seeding. In March of each year, about 60,000 seeds were sown at each plot, the lands were plowed to a depth of 20 cm, and no irrigation was applied during the growing seasons. Approximately 250 plant m^−2^ successfully matured. Crops were harvested in September, thereby leaving the lands under low cover for 5–6 months per year. All other field management activities were performed according to local farming habits.

### Rhizosphere Soil Collection and Physicochemical Analyses

Rhizosphere soil samples were collected from fields with continuously monocropped Tibetan barley for 2–6 years (encoded as CC2Y, CC3Y, CC4Y, CC5Y, and CC6Y) annually in June during the flowering period. Approximately 15 plant samples were collected from five different sites using a “Z” pattern in each experimental plot. A composite sample was produced from the 15 plants. Rhizosphere soils were collected for analysis as previously reported (Li et al., 2018). Briefly, plants were carefully pulled from the ground and the conglutinating soil from the roots was mildly crushed and shaken to collect the soil located within the plant root surfaces. Quadruple soil and plant samples were collected from the four experimental fields. The rhizosphere soil was immediately transported on ice to the laboratory and stored at −20°C for DNA extraction and chemical analysis. Half of each combined soil sample was air-dried and filtered through a 0.149-mm sieve. The sample pH was determined using a pH meter with a glass electrode (FE20-Five Easy Plus™, Mettler Toledo, Greifensee, Switzerland). Total nitrogen (TN), available nitrogen (AN), total phosphorus (TP), total potassium (TK), rapidly available phosphorus (RAP), and rapidly available potassium (RAK) were measured as previously described (Nichols and Wright, 2006).

### Soil DNA Extraction, PCR Amplification, and Illumina MiSeq Sequencing

Community DNA was extracted using a MoBio™ Soil DNA isolation kit (MO BIO Laboratories Inc., MA, United States) according to the manufacturer’s protocol. The DNA concentration and purity were determined with a NanoDrop 2000 Spectrophotometer (Thermo Scientific, United States). The 18S rRNA gene was amplified using the universal eukaryotic primer pair NS1 (5′-GTA GTC ATA TGC TTG TCT C-3′) and Fung (5′-ATT CCC CGT TAC CCG TTG-3′; Möhlenhoff et al., 2001) with a 12-base unique barcode attached at the forward primer. The PCR reactions and amplicon purification were performed as described by Cheung et al. (2010). The PCR products from the three replicate amplifications were separately pooled and evaluated on 2% agarose gels. Finally, the purified products were quantified by a Qubit3.0 (Thermo Fisher Scientific, United States) and then sequenced on an Illumina MiSeq platform (Sangon Biotech Co., Ltd., Shanghai, China), whereupon 250-bp paired-end reads were generated.

### Processing of Sequencing Data

The Quantitative Insights into Microbial Ecology (QIIME 2) software (Bolyen et al., 2019) was used to process the 18S rRNA gene sequences after the paired-end fragments were merged by FLASH (version 1.2.11; Magoč and Salzberg, 2011). Briefly, the demux plugin^1^ was used to demultiplex and classify the sequences into the different samples based on the unique barcode. Then, the q2-feature-classifier plugin^2^ was used for quality control and chimeric sequence removal with the default settings. After that, UCLUST (version 1.2.22; Edgar, 2010) was applied to cluster the sequences into operational taxonomic units (OTUs) at 97% similarity. The classifier–sklearn plugin^3^ was used for the taxonomic assignment of each sequence with the SILVA123 reference database (Quast et al., 2012). A total of 783,935 high-quality sequences of 18S rRNA were obtained. The OTU table was rarefied to the minimum sample count (38,031 sequences for each sample) to reduce the effect of sequence numbers on diversity calculations. The alpha diversity was calculated, in which Chao1 was used to describe the fungal species richness, and the PD index was used to determine the phylogenetic diversity (Cadotte et al., 2012; Thukral, 2017). To compare the fungal community beta diversity, principal coordinates analysis (PCoA), based on the Bray–Curtis distance metric, was performed using the “phyloseq” package (McMurdie and Holmes, 2013) and plotted by the “ggplot2” package (Wickham et al., 2016). Taxonomic classification was conducted with the SILVA123 reference database for fungi (Quast et al., 2012).

### Co-occurrence Network Construction and Keystone OTU Identification

A prepared OTU file containing OTUs with relative abundances higher than 0.1% and only occurring in all rhizosphere soil samples from individual cropping years was used. Individual fungal networks within the samples from 2 to 6 years of continuous cropping were constructed. To describe the complex pattern of fungal taxon interactions, a set of network topological properties was calculated using the “igraph” package in R (Csardi and Nepusz, 2006). Network visualization was performed using the interactive platform Gephi (Bastian et al., 2009). In addition, keystone OTUs were identified from the co-occurrence network. The OTUs with the highest degree, highest closeness centrality, and lowest betweenness centrality scores were considered as the keystone taxa (Banerjee et al., 2018). For the overall network, the keystone taxa comprised OTUs with degree >90, closeness centrality >0.7, and betweenness centrality lower than 0.5. For phylogenetic analysis of the keystone OTUs, representative sequences belonging to the OTUs were extracted using QIIME and taxonomically classified after re-alignment with the SILVA123 reference database (Quast et al., 2012).

### Statistical Analysis

Statistical analyses were conducted using SPSS 18.0 (SPSS Inc., Chicago, IL, United States). Statistical significance was estimated with one-way ANOVA followed by Tukey’s honestly significant difference (HSD) *post hoc* test to determine the significance of the differences between groups. Additionally, three complementary non-parametric multivariate statistical tests, that is, Adonis (non-parametric MANOVA), ANOSIM (analysis of similarity), and MRPP (multi-response permutation procedure), were used to compare the microbial community differences of two cropping years using the Bray–Curtis distance with 999 permutations, which was performed in R using the “vegan” package. Differences were considered to be significant when the value of *p* was less than 0.05. Correlations between yields and environmental variables were estimated by Spearman’s correlation coefficients.

### Nucleic Acid Sequences

The 18S rRNA gene sequences were deposited into the NCBI Sequence Read Archive with the accession number PRJNA669607.

## Results

### Relationships Between Environmental Parameters and Fungal Community Diversity

Total precipitation during the growing seasons (March through September of the following year) ranged from 394.9 to 596.3 mm (Supplementary Table S1). Furthermore, the recorded temperature during the growing seasons did not exhibit significant changes, with an average monthly temperature of 7.41–8.99°C. However, the plant height at the time of harvest decreased from 116.73 ± 1.37 to 98.33 ± 2.77 cm after 6 years of Tibetan barley monoculture. Moreover, the yields consistently decreased, from 19.42 ± 0.74 to 12.40 ± 0.45 kg ha^−1^ (Supplementary Table S1). No significant relationship was observed between yields and precipitation (with a Spearman correlation coefficient of −0.26, *p* = 0.62) or temperature (with a Spearman correlation coefficient of −0.60, *p* = 0.21).

**Figure 1:**
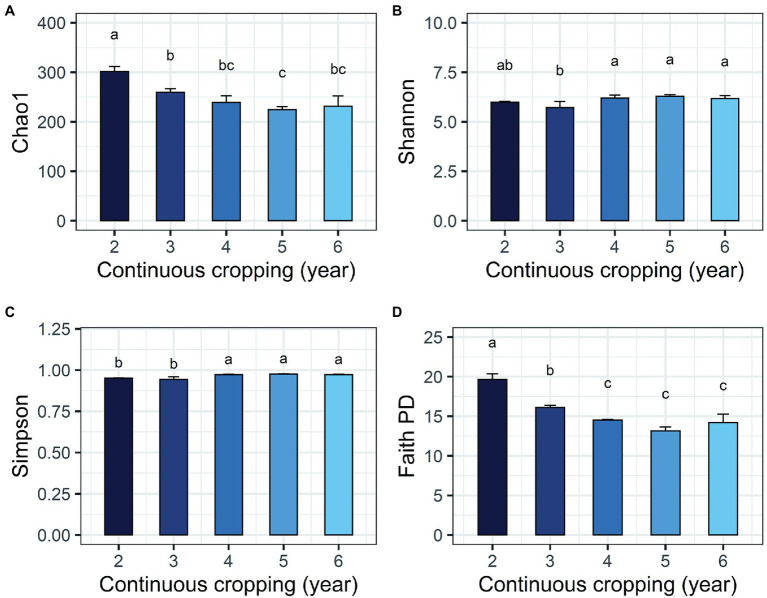
Changes in alpha diversity indices of the fungal community during continuous cropping of Tibetan barley. **(A)** Chao1; **(B)** Shannon; **(C)** Simpson; **(D)** PD indices. Averages ± SD of samples in each group (with four biological replicates) are expressed in each column. Different letters within a row indicate significant differences at *p* < 0.05.

**Figure 2:**
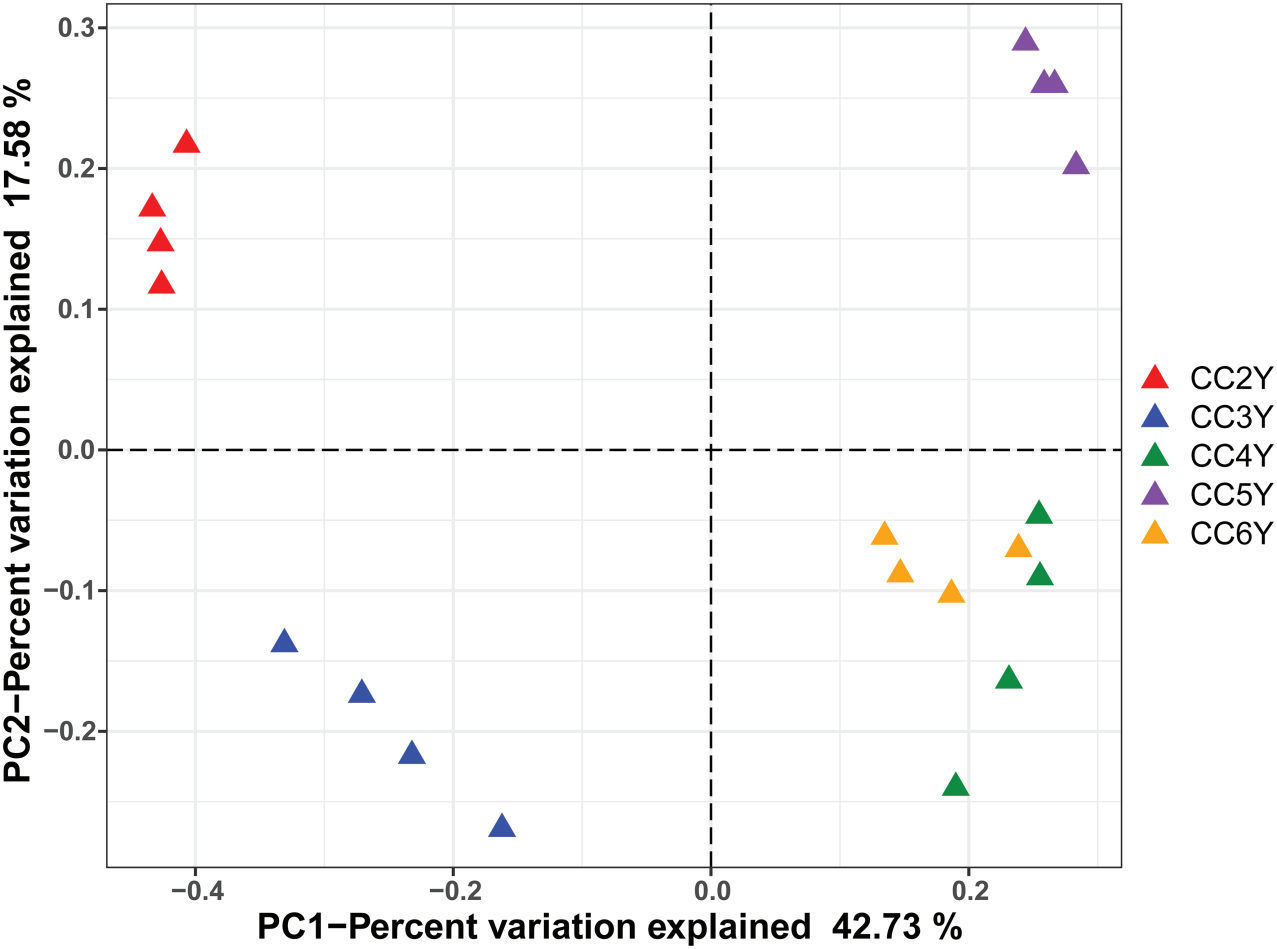
PCoA of fungal communities in the rhizosphere soil samples based on Bray–Curtis distances. CC2Y, CC3Y, CC4Y, CC5Y, and CC6Y represent continuous cropping for 2, 3, 4, 5, and 6 years, respectively. The percent variation of the plotted principal component is indicated on the axes.

The environmental parameters of the rhizosphere soil were influenced by continuous cropping, although rhizosphere soil pH exhibited no obvious changes throughout the study and was slightly alkaline (pH ranged from 8.06 to 8.91; Supplementary Table S2). The concentration of TN and AN decreased with continuous cropping. However, phosphorus (TP and RAP) and potassium (TK and RAK) fluctuated and did not exhibit increasing or decreasing trends throughout the experimental period (Supplementary Table S2). Continuous cropping negatively affected the species richness and phylogenetic diversity of the rhizosphere fungal communities of Tibetan barley (Figure 1). The Chao1 and PD indices of the rhizosphere fungal community significantly declined from 2 to 5 years, but slightly increased after 5 years of continuous cropping. The Chao1 index decreased from 301.5 ± 10.23 at 2 years to 224.8 ± 6.6 at 5 years of continuous cropping and slightly climbed to 233.0 ± 21.3 after 6 years. The PD index decreased from 19.8 ± 0.6 to 13.4 ± 0.5 and slightly increased to 14.5 ± 1.1 after 6 years. However, the Simpson and Shannon indices did not exhibit decreasing trend during 6 years of continuous cropping (Figure 1). PCoA was used to depict the degree of fungal community differentiation from different durations of continuous cropping. The first two principal coordinates explained 60.31% of the total variance of the rhizosphere soil fungal community structures (Figure 2). All soil samples were well separated from each other, indicating that fungal community structures were significantly different among the treatments. PCoA revealed that fungal communities from the second and 3rd years (CC2Y and CC3Y) were more separated from those from later years (CC4Y, CC5Y, and CC6Y) along the first PCoA axis. Communities from the last 3 years were separated along the second PCoA axis (Figure 2). Three complementary non-parametric multivariate statistical tests (Adonis, ANOSIM, and MRPP) all showed that the community structure was significantly different among treatments (*p* < 0.05; Supplementary Table S3).

### Changes in Fungal Community Composition With Continuous Cropping Duration

**Figure 3:**
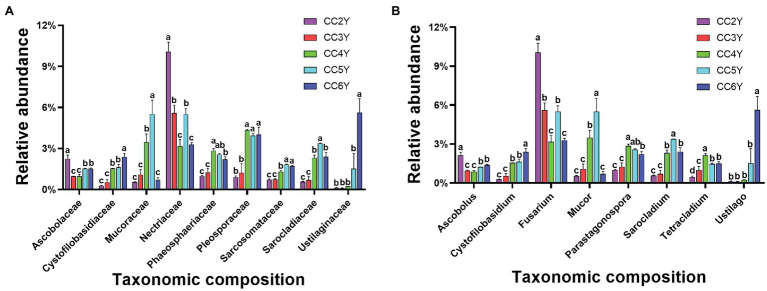
Relative abundance of fungal taxa in the rhizosphere soil of Tibetan barley continuously cropped for different durations. **(A)** Relative abundances of the 10 most abundant fungal families. **(B)** Relative abundances of the 10 most abundant fungal genus. Symbols represent means (*n* = 4).

Nearly 79% of the total sequences of the 18S rRNA gene could be taxonomically classified at the phylum level. *Ascomycota* (56.74% ± 4.91%), *Basidiomycota* (17.45% ± 7.60%), and *Mucoromycota* (3.21% ± 1.91%) were the dominant phyla (Supplementary Figure S1), accounting for more than 75% of all sequences. The predominant families in the rhizosphere soil were *Nectriaceae*, *Mucoraceae*, and *Pleosporaceae* (Figure 3A). *Nectriaceae* was the only abundant family that exhibited a decreasing trend, from 10.06% ± 0.71% to 3.28% ± 0.14%, throughout the experimental duration. Yet, the abundances of other predominated families all exhibited increasing patterns during continuous cropping, including *Cystofilobasidiaceae*, *Mucoraceae*, *Phaeosphaeriaceae*, *Pleosporaceae*, *Sarcosomataceae*, *Sarocladiaceae*, and *Ustilaginaceae*. Among them, *Ustilaginaceae* increased 46.8-fold from 0.12% ± 0.02% to 5.62% ± 1.02% (Figure 3A). As shown in Figure 3B, at the genus level, *Fusarium*, *Mucor*, *Parastagonospora*, *Sarocladium*, and *Ustilago* mostly appeared across all samples. The relative abundance of *Fusarium* fluctuated and tended to decrease from 10.06% ± 0.71% to 3.28% ± 0.14% with the extension of cropping years. In addition, *Cystofilobasidium* and *Ustilago* increased after 6 years of continuous cropping.

**Figure 4:**
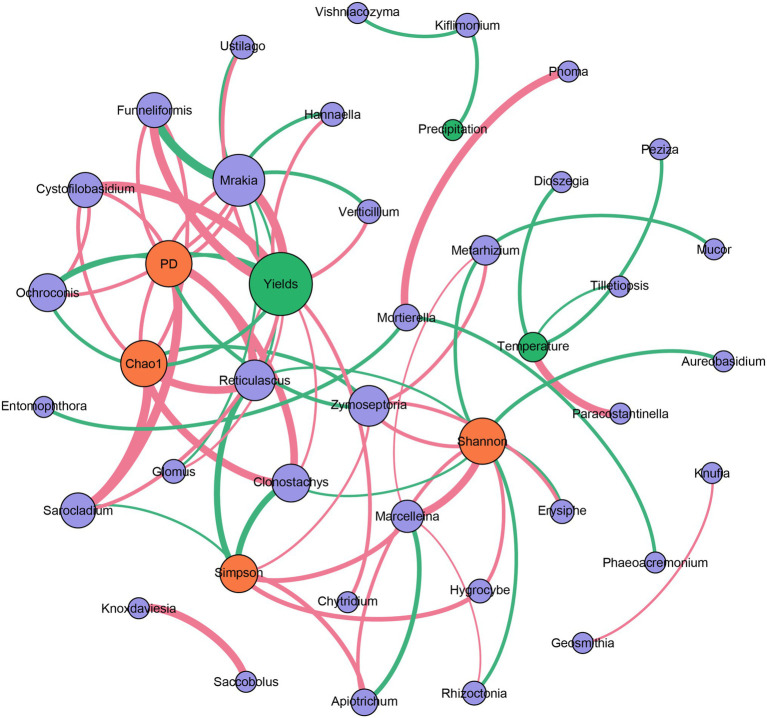
Correlation-based network analysis showing potential interactions between fungal community (purple circle: genera; orange circle: alpha diversity indices) and environmental variables (green circle). A connection indicates a strong (|*r*| of >0.6) and significant (*p* of <0.05) Spearman’s correlation. Red lines indicate negative correlations, while green lines indicate positive correlations. The thickness of each edge is proportional to the value of Spearman’s correlation.

The correlation-based network analysis revealed that alpha diversities of the fungal community (Chao1 and PD indices) were all negatively related to the yields of Tibetan barley (*p* < 0.05). For genus composition, negative relationships were observed between *Ochroconis* and yields. However, a significant relationship was not observed between temperature and the alpha diversities of the fungal community, nor between precipitation and these indices. In addition, many other genera were positively related to the yields of Tibetan barley (all *p* < 0.05), and the highest Spearman’s correlation coefficients were from *Mrakia*, *Funneliformis*, and *Cystofilobasidium*. Furthermore, only a small number of genera have a significant relationship with temperature and precipitation (Figure 4). We also explored the changes in 20 most abundant OTUs during continuous cropping. The abundance of OTU1 (classified as unclassified *Capnodiales*), OTU8 (*Parastagonospora* sp.), OTU10 (Unclassified *Xylariales*), OTU11 (*Sarocladium* sp.), OTU12 (Unclassified *Pleosporaceae*), OTU13 (Unclassified *Tremellales*), OTU15 (Unclassified *Fungi*), OTU16 (*Ustilago* sp.), and OTU20 (*Cystofilobasidium* sp.) increased significantly during 6 years of continuous cropping. By contrast, the OTUs decreased significantly, including OTU2 (unclassified *Hypocreales*), OTU3 (unclassified *Fungi*), OTU4 (*Fusarium oxysporum*), and OTU17 (unclassified *Fungi*; Supplementary Table S4).

### Changes in the Keystone OTUs of Fungal Communities

Co-occurrence networks at the OTU level were constructed to reveal the interactions of fungal species within the samples of Tibetan barley continuously cropped for different durations (Supplementary Figure S2). The resulting complex pattern of the relationships between nodes was depicted by computing the topological properties (Supplementary Table S5). The number of nodes (OTUs) decreased from 126 to 108, while the edges (connections) increased from 242 to 863 during the 6 years of Tibetan barley continuous cropping. Increased ratio of edges to nodes (1.921–7.991) and network density (from 0.031 to 0.149) was also identified. Considering the potential functioning of the keystone fungal group underlying the continuous cropping of Tibetan barley, we analyzed the keystone OTUs in each co-occurrence network from different years of continuous cropping. Keystone OTUs with the highest degree, highest closeness centrality, and lowest betweenness centrality scores were represented by the larger circle size in Supplementary Figure S2. We found that most of the keystone OTUs belonged to the phyla *Ascomycota* and *Basidiomycota*, which were affiliated with *Agaricomycetes*, *Dothideomycetes*, *Eurotiomycetes*, *Orbiliomycetes*, *Pezizomycetes*, *Sordariomycetes*, and *Tremellomycetes* at the class level (Supplementary Table S6). Notably, OTU39 and OTU61, in the rhizosphere soils of CC2Y, were affiliated with the order *Pleosporales* and unclassified Fungi. In contrast, the numbers of keystone OTUs were highest in CC3Y and CC4Y, and some OTUs could be classified at the species level, including OTU47 (classified as *Verticillium alfalfae*), OTU85 (*Clonostachys rosea*), and OTU136 (*Geosmithia putterillii*). Furthermore, four and three keystone OTUs were identified in CC5Y and CC6Y networks, respectively, and these OTUs were classified into the genera *Cystofilobasidium*, *Kiflimonium*, and *Knufia*.

## Discussion

Plants and microbes have co-evolved and interact with each other in the environment. High-altitude ecosystems are generally characterized by low temperature, variable rainfall, low atmospheric pressure, and soil nutritional stress. Cold in high-altitude regions is a major factor that has confounded effects on both microbial biodiversity and soil physicochemical properties (Kumar et al., 2019). Thus, high-altitude regions are not only the hotspots of diverse groups of microbes but also the research center in understanding the influences of certain microbes on the growth and productivity of the plants cultivated in cold environments (Rawat et al., 2020). As “the third pole” on Earth, Qinghai–Tibetan Plateau is attracting researchers interested in understanding the response of microbes to environmental changes. Previous studies suggested that climate change and human activities altered the diversity and composition of soil microbial communities in the alpine grasslands of the Qinghai–Tibetan Plateau (Zhang et al., 2016a). Furthermore, the influence of phosphorus and nitrogen addition on the composition of the soil fungal community in the alpine meadow on the Qinghai–Tibetan Plateau has been explored (He et al., 2016). However, root-associated fungi link resource fluxes between the soil and roots, thus influencing plant growth and ecosystem function. Despite this, the fungal community structure of rhizosphere soils in the high-altitude region is not deeply understood. Agrotype, climate change, and human activities are the main factors in controlling the community structure and functions of rhizosphere soil fungi (Frąc et al., 2018). The fungal communities of rhizosphere soil of Tibetan barley must be tolerant of drought and cold, and Tibetan barley is adaptive to the extreme climate of the Qinghai–Tibetan Plateau. We identified *Fusarium*, *Mucor*, *Parastagonospora*, *Sarocladium*, and *Ustilago*, which mostly appeared across all rhizosphere soil samples during the continuous cropping of Tibetan barley. These abundant genera consisted of various pathogen species, causing pathogenicity as well as limited crop yield in a wide range of plants (Wakelin et al., 2008). In particular, *Fusarium* has always been identified as a link to soil sickness during the continuous cropping of cucumber and soybean (Zhou and Wu, 2012; Liu et al., 2019). In addition, *Ustilago*, another soil-borne potential plant pathogen, increased after continuous soybean cropping (Liu et al., 2019).

Environmental variables have strong influences on microbial community structure in rhizosphere soils. It was observed that the community richness and diversity of the fungal community in the rhizosphere soils of *Robinia pseudoacacia* L. increased when exposed to elevated air temperature (Jia et al., 2019). Elevated air temperature causes clear shifts in rhizosphere microbial community structure, possibly through effects on plant growth. The temperature could enhance the release of the exudates from roots, leading to changes in the microbial community (Jia et al., 2019). In addition, as an another key environmental factor, precipitation is a driving force in shaping soil fungal community structures, and it has been reported that precipitation shaped the communities of arbuscular mycorrhizal fungi in Tibetan alpine steppe (Zhang et al., 2016b). However, in our study, total precipitation during the growing seasons fluctuated during the studied years, and the recorded temperature did not exhibit significant changes (Supplementary Table S1). In addition, correlation analysis indicated that air temperature and precipitation have little influence on the fungal communities in the rhizosphere soil during the continuous cropping of Tibetan Barley (Figure 4). The influences of temperature and precipitation on the fungal community of rhizosphere soil should be dependent on the physiological property of the plant. Tibetan barley is drought- and cold-tolerant, exhibiting strong adaptability to the extreme climate of the plateau (Zeng et al., 2018). Thus, the fungal community in the rhizosphere of Tibetan barley may be not sensitive to air temperature and precipitation. Although no significant relationship was observed between yields and precipitation or air temperature in this study, the alpha diversities of the fungal community were all negatively related to the yields of Tibetan barley, and many relationships were observed between fungal genera and yields (Figure 4). These results indicated that the fungal community in rhizosphere soils responds to the continuous cropping of Tibetan barley but not to other environmental variables, such as temperature and precipitation.

Decreases in fungal richness have been recognized as a grave threat to the ecosystem and may hurt plant production during continuous cropping (Singh et al., 2014; Maron et al., 2018). Negative relationships between alpha diversity indices and cropping years results have also been observed in the rhizosphere soils of continuously monocropped tea (Arafat et al., 2019), American ginseng (Dong et al., 2017), and sweet potato (Gao et al., 2019). In this study, Chao1 and PD indices of the fungal community were significantly negatively correlated with cropping year (Figure 1). However, the Simpson and Shannon indices did not exhibit any decreasing tend during the 6 years of continuous cropping, indicating that the continuous cropping of Tibetan barley influenced species richness and phylogenetic diversities of fungal communities, other than evenness.

The effects of continuous cropping on soil fungal communities (especially community diversity and taxonomic composition) have been intensively studied; however, not much knowledge is available on the network structure and interactions among the fungal species affected by continuous cropping in rhizosphere soils. The results showed that the continuous cropping of Tibetan barley induces a small size with fewer nodes but more links on the fungal co-occurrence network (Supplementary Table S5; Supplementary Figure S2). The increasing connectivity (number of edges) and decreasing hubs (number of nodes) suggested that the fungal network of nodes affected by each other could be more efficient or intense under long-term continuous cropping (He et al., 2017). In this study, we also observed that the values of modularity and network heterogeneity decreased with the extension of continuous cropping years, suggesting that continuous cropping induced more unstable network structures in the fungal communities in the rhizosphere soil (Supplementary Table S5). The decreased instability was probably due to fungal diversity loss and community structure simplifications caused by long-term continuous cropping. In summary, an increased continuous cropping duration may result in the accumulation of environmental stress, which would destabilize fungal community networks.

The microorganisms coexisting in complex networks offer insights into the underlying properties of the microbial network structures and their putative keystone species, indicating their ecological processes (Barberán et al., 2012). Besides, keystone species are considered responsible for maintaining ecosystem functions and play important roles in biogeochemical cycling (Lynch and Neufeld, 2015). The keystone OTUs in the fungal network were representatives of *Pleosporales*, *Hypocreales*, and *Chaetothyriales* at the order level. *Pleosporales* appear to play pivotal functions in decomposing plant residues in the soil (Wang et al., 2014). As the most common keystone genus in the network, *Knufia* has been reported as a typical rock-inhabiting black fungus that could corrode extracellular polysaccharides (Breitenbach et al., 2018). This specific character may help *Knufia* thrive in drought habitats in the rhizosphere soil of Tibetan barley. Members of *Cystofilobasidium* have psychrophilic abilities (Nakagawa et al., 2005), and this allows them to extensively colonize the rhizosphere soil of Tibetan barley cultivated on the Qinghai–Tibetan Plateau with a low average annual temperature (about 1°C). *Clonostachys rosea* is a promising saprophytic filamentous fungus exhibiting strong biological control ability against numerous fungal plant pathogens (Sun et al., 2020). It has been proved that *C. rosea* is an effective bioagent in controlling pathogens causing root rot complex in field pea (Xue, 2003). The antagonistic activity of *C. rosea* to the rhizosphere pathogens may be used to reduce the effect of accumulated pathogenic abundance on plant roots, which is a common phenomenon after the long-term continuous monocropping of plants. Collectively, these keystone OTUs had the most interactions with other fungi and may control the co-occurrence network pattern of fungal communities during the continuous cropping of Tibetan barley. However, their response to continuous cropping and their roles in the rhizosphere soil of Tibetan barley are ambiguous and need to be explored.

## Conclusion

In summary, our results revealed that continuous cropping exerted substantial impacts on the fungal diversity, co-occurrence network structures, as well as keystone species of rhizosphere soil continuously cropped with Tibetan barley. Specifically, continuous cropping markedly reduced the fungal richness and phylogenetic diversity. Furthermore, increased proportions of the nodes had positive edges, and decreasing network modularity and heterogeneity were observed during the extension of continuous cropping years. Additionally, continuous cropping also altered the keystone OTUs from different years of continuous cropping. Overall, our research has contributed to the knowledge of the response of fungal networks to the continuous cropping of plants cultivated in high-altitude areas, but the interactions of fungal species in the rhizosphere during continuous cropping should be further examined.

## Data Availability Statement

The datasets presented in this study can be found in online repositories. The names of the repository/repositories and accession number(s) can be found at: https://www.ncbi.nlm.nih.gov/, PRJNA669607.

## Author Contributions

YY and YZ carried out the molecular experiments, analyzed the data, and wrote the manuscript. XY and LA carried out data analyses. YB and XL contributed to the field experiments and collected the samples. KW conceived the study, contributed to the design, and interpreted the research. All authors read and approved the final manuscript.

## Funding

This work was supported by the National Key R&D Program of China (grant no. 2019YFD1001700) and the National Science Foundation of China (grant no. 32060447).

## Conflict of Interest

The authors declare that the research was conducted in the absence of any commercial or financial relationships that could be construed as a potential conflict of interest.

## Publisher’s Note

All claims expressed in this article are solely those of the authors and do not necessarily represent those of their affiliated organizations, or those of the publisher, the editors and the reviewers. Any product that may be evaluated in this article, or claim that may be made by its manufacturer, is not guaranteed or endorsed by the publisher.

## Footnotes

^1^ https://github.com/qiime2/q2-demux

^2^ https://github.com/qiime2/q2-dada2

^3^ https://github.com/qiime2/q2-feature-classifier

## Supplementary Material

Supplementary Figure S1 Relative abundance of fungal phyla in the rhizosphere soil of Tibetan barley continuously cropped for different durations. Values represent means ± SD (*n* = 4). Different lowercase letters within the same column indicate significant differences among different continuous cropping durations at *p* < 0.05 according to a two-way ANOVA.

Supplementary Figure S2 Co-occurrence networks show the correlations among total fungal OTUs in CC2Y **(A)**, CC3Y **(B)**, CC4Y **(C)**, CC5Y **(D)**, and CC6Y **(E)**. A connection indicates a strong (|*r*| of ≥ 0.8) and significant (*p* of ≤ 0.01) Spearman’s correlation. Red lines indicate positive correlations, while green lines indicate negative correlations. The network is colored by a module that clusters the tightly connected nodes.
