# Supplementary material for: Impacts of Continuous Cropping on Fungal Communities in the Rhizosphere Soil of Tibetan Barley
Source: Front Microbiol. 2022 Feb 4;13:755720. doi: 10.3389/fmicb.2022.755720 (PMC8854972; doi:10.3389/fmicb.2022.755720)
Supplement: Supplementary file 1 [file Data_Sheet_1.docx]

**Supplementary Tables for**

**Continuous Cropping Reduces the Diversity and Simplifies the Co-occurrence Network of Fungal Communities in the Rhizosphere Soil of Tibetan Barley**

Youhua Yao^1,2,3,#^, Yuan Zhao^4,#^, Xiaohua Yao^1,2,3^, Yixiong Bai^1,2,3^, Likun An^1,2,3^, Xin Li^1,2,3^, Kunlun Wu^1,2,3,*^

^1^ Academy of Agricultural and Forestry Sciences, Qinghai University, Xining, China.

^2^ Qinghai Key Laboratory of Hulless Barley Genetics and Breeding, Xining, China.

^3^ Qinghai Subcenter of National Hulless Barley Improvement, Xining, China.

^4^ School of Ecological and Environmental Engineering, Qinghai University, Xining, China.

^#^ These authors contributed equally to this work.

∗Correspondence to: KL Wu, Academy of Agricultural and Forestry Sciences, Qinghai University, Xining 810016, China. E-mail: wklqaaf@sina.com;

**Table S1**. Changes in meteorology and plant during continuous cropping of Tibetan Barley.

| **Year** | **Precipitation (mm)^a^** | **Temperature (^o^C)^b^** | **Plant Height (cm)** | **Yields (kg ha^-1^)** |
| --- | --- | --- | --- | --- |
| 2011 | 446.6 | 7.41 | 116.73±1.37 a | 19.42±0.74 a |
| 2012 | 456.6 | 7.85 | 108.87±7.25 abc | 17.60±1.15 a |
| 2013 | 394.9 | 8.71 | 109.40±6.20 ab | 16.53±1.00 b |
| 2014 | 596.3 | 7.94 | 103.97±2.93 bc | 15.24±0.62 bc |
| 2015 | 428.1 | 7.83 | 100.10±3.32 bc | 13.47±0.39 cd |
| 2016 | 544.7 | 8.99 | 98.33±2.77 c | 12.40±0.45 d |

^a^ The values were shown as the total precipitations during the growing seasons (March through September of the following year).

^b^ The values were shown as the average monthly temperature during the growing seasons.

^c^ Different letters within a row indicate significant differences at *P* < 0.05.

**Table S2**. Effects of continuous cropping on rhizosphere soil physicochemical characteristics.

| Physicochemical factors | Duration of continuous cropping (years) | | | | |
| --- | --- | --- | --- | --- | --- |
|  | 2 | 3 | 4 | 5 | 6 |
| pH | 8.42±0.38 | 8.45±0.15 | 8.52±0.31 | 8.36±0.10 | 8.57±0.22 |
| TP (g·kg^−1^) | 1.63±0.10**^c^** | 2.26±0.13**^a^** | 1.97±0.11**^b^** | 2.26±0.07**^a^** | 1.92±0.05**^b^** |
| TK (g·kg^−1^) | 20.14±0.33**^c^** | 22.85±0.59**^ab^** | 22.01±0.35**^b^** | 23.26±0.45**^a^** | 22.95±0.70**^ab^** |
| AN (mg·kg^−1^) | 194.25±5.12**^a^** | 189.25±8.42**^a^** | 118.75±3.50**^b^** | 130.00±3.74**^b^** | 88.75±4.03**^c^** |
| RAP (mg·kg^−1^) | 50.15±1.92**^c^** | 97.68±3.31**^a^** | 53.83±1.30**^c^** | 98.05±2.95**^a^** | 68.90±6.74**^b^** |
| RAK (mg·kg^−1^) | 74.18±3.97**^b^** | 80.33±2.49**^ab^** | 79.78±9.04**^abc^** | 87.78±4.31**^a^** | 61.43±3.01**^c^** |

Values represent means ± standard deviation (n = 4). Different lowercase letters within the same column indicate significant differences among different continuous cropping durations at *P* < 0.05 according to Duncan’s test.

TN, total nitrogen; TP, total phosphorus, TK, total potassium; AN, available nitrogen; RAP, rapidly available phosphorus; RAK, rapidly available potassium.

**Table S3**. The significant difference between different groups is based on the Bray–Curtis metric distance determined by Adonis, ANOSIM, and MRPP.

| Treatment | | Three complementary non-parametric multivariate statistical tests | | |
| --- | --- | --- | --- | --- |
|  |  | Adonis | ANOSIM | MRPP |
| CC6Y | CC3Y | 0.026 | 0.026 | 0.03 |
| CC6Y | CC5Y | 0.031 | 0.029 | 0.032 |
| CC6Y | CC2Y | 0.03 | 0.036 | 0.026 |
| CC6Y | CC4Y | 0.028 | 0.038 | 0.035 |
| CC3Y | CC5Y | 0.028 | 0.031 | 0.033 |
| CC3Y | CC2Y | 0.029 | 0.032 | 0.041 |
| CC3Y | CC4Y | 0.026 | 0.031 | 0.032 |
| CC5Y | CC2Y | 0.025 | 0.031 | 0.033 |
| CC5Y | CC4Y | 0.03 | 0.032 | 0.025 |
| CC2Y | CC4Y | 0.027 | 0.029 | 0.026 |

Differences between groups were considered significant when *P* ≤ 0.05.

**Table S4**. The changes of the 20 most abundant OTUs during continuous cropping.

| **OTU ID** | **Taxonomic assignment** | **Relative abundance *** | | | | |
| --- | --- | --- | --- | --- | --- | --- |
|  |  | **CC2Y** | **CC3Y** | **CC4Y** | **CC5Y** | **CC6Y** |
| OTU1 | Unclassified *Capnodiales* | 4.69%±0.23%c | 4.59%±0.90%c | 8.93%±0.62%a | 6.54%±0.83%b | 9.25%±1.17%a |
| OTU2 | Unclassified *Hypocreales* | 9.30%±1.06%b | 12.59%±1.97%a | 2.56%±0.07%c | 2.62%±0.07%c | 5.40%±2.26%c |
| OTU3 | Unclassified Fungi | 15.87%±0.69%a | 16.10%±3.19%a | 0.07%±0.01%b | 0.09%±0.01%b | 0.08%±0.03%b |
| OTU4 | *Fusarium oxysporum* | 8.90%±0.63%a | 4.97%±0.53%b | 2.68%±0.54%c | 5.04%±0.46%b | 2.66%±0.16%c |
| OTU5 | Unclassified *Pleosporales* | 3.72%±0.57%a | 1.59%±0.66%b | 4.42%±0.03%a | 4.47%±0.21%a | 3.66%±0.28%a |
| OTU6 | *Mucor endophyticus* | 0.54%±0.03%c | 1.08%±0.38%c | 3.47%±0.59%b | 5.51%±1.02%a | 0.71%±0.18%c |
| OTU7 | Unclassified *Basidiomycota* | 0.00%±0.00%b | 1.92%±1.64%b | 8.41%±1.60%a | 0.00%±0.00%b | 0.03%±0.06%b |
| OTU8 | *Parastagonospora* sp. | 0.99%±0.05%c | 1.24%±0.30%c | 2.84%±0.14%a | 2.57%±0.06%ab | 2.21%±0.19%b |
| OTU9 | Unclassified *Agaricomycetes* | 1.35%±0.20%bc | 0.41%±0.13%c | 1.85%±0.88%b | 5.31%±1.06%a | 0.46%±0.09%c |
| OTU10 | Unclassified *Xylariales* | 0.00%±0.00%c | 0.46%±0.39%c | 1.97%±0.33%b | 1.33%±0.89%bc | 4.95%±0.91%a |
| OTU11 | *Sarocladium* sp. | 0.57%±0.04%c | 0.57%±0.19%c | 1.88%±0.30%b | 3.27%±0.04%a | 2.19%±0.26%b |
| OTU12 | Unclassified *Pleosporaceae* | 0.64%±0.05%b | 0.70%±0.30%b | 2.28%±0.03%a | 2.32%±0.05%a | 2.17%±0.32%a |
| OTU13 | Unclassified *Tremellales* | 0.66%±0.04%c | 0.93%±0.32%c | 2.15%±0.10%ab | 1.82%±0.14%b | 2.23%±0.15%a |
| OTU14 | Unclassified *Agaricomycetes* | 0.23%±0.06%b | 1.07%±0.57%b | 3.23%±0.59%a | 0.75%±0.39%b | 2.39%±0.39%a |
| OTU15 | Unclassified Fungi | 0.47%±0.35%c | 1.88%±0.27%ab | 0.99%±0.21%bc | 1.80%±0.10%ab | 2.49%±1.08%a |
| OTU16 | *Ustilago* sp. | 0.12%±0.02%b | 0.09%±0.03%b | 0.24%±0.01%b | 1.53%±1.12%b | 5.62%±1.02%a |
| OTU17 | Unclassified Fungi | 1.19%±1.03%b | 5.21%±1.05%a | 0.00%±0.00%b | 0.00%±0.00%b | 0.00%±0.00%b |
| OTU18 | *Tetracladium marchalianum* | 0.40%±0.06%d | 0.99%±0.28%cd | 2.10%±0.13%a | 1.40%±0.06%b | 1.50%±0.13%bc |
| OTU19 | Unclassified *Tremellomycetes* | 0.54%±0.02%d | 0.77%±0.34%c | 2.42%±0.19%a | 1.51%±0.06%b | 1.14%±0.06%b |
| OTU20 | *Cystofilobasidium* sp. | 0.28%±0.01%c | 0.54%±0.20%c | 1.54%±0.00%b | 1.63%±0.21%b | 2.39%±0.25%a |

* Relative abundance are represented as average ± SD obtained across quadruple measurements. Average with different superscript letters are significantly different horizontally (*P* < 0.05).

**Table S5**. Topological characteristics of the co-occurrence network of the fungal communities for the rhizosphere soils of continuously cropped Tibetan barley.

| **Topological properties** | **Duration of continuous cropping (years)** | | | | |
| --- | --- | --- | --- | --- | --- |
|  | 2 | 3 | 4 | 5 | 6 |
| Number of nodes | 126 | 114 | 117 | 117 | 108 |
| Number of edges | 242 | 304 | 406 | 934 | 863 |
| Ratio of edges to nodes | 1.921 | 2.667 | 3.470 | 7.983 | 7.991 |
| Percentage of positive edges | 35.90% | 43.75% | 53.94% | 49.89% | 53.77% |
| Percentage of negative edges | 64.10% | 56.25% | 46.06% | 50.11% | 46.23% |
| Modularity | 0.51 | 0.22 | 0.34 | 0.49 | 0.35 |
| Network density | 0.031 | 0.047 | 0.060 | 0.138 | 0.149 |
| Network heterogeneity | 2.94 | 2.861 | 2.529 | 1.495 | 1.538 |
| Network centralization | 0.717 | 0.853 | 0.851 | 0.702 | 0.781 |

**Table S6**. Information of the keystone OTUs of fungal networks in rhizosphere soils under the continuous cropping of Tibetan barley.

| **Network ID** | **OTU ID** | **Betweenness centrality** | **Closeness centrality** | **Node degree** | **Taxonomic information** | | | | | |
| --- | --- | --- | --- | --- | --- | --- | --- | --- | --- | --- |
|  |  |  |  |  | **Phylum** | **Class** | **Order** | **Family** | **Genus** | **Specie** |
| CC2Y | OTU39 | 0.49 | 0.95 | 92 | *Ascomycota* | *Dothideomycetes* | *Pleosporales* | |  |  |
|  | OTU61 | 0.49 | 0.95 | 92 | unclassified Fungi |  |  |  |  |  |
| CC3Y | OTU201 | 0.09 | 0.87 | 96 | unclassified Fungi |  |  |  |  |  |
|  | OTU34 | 0.09 | 0.87 | 96 | *Ascomycota* |  |  |  |  |  |
|  | OTU58 | 0.09 | 0.87 | 96 | *Ascomycota* | *Pezizomycetes* | *Pezizales* | *Pezizaceae* | | uncultured *Pezizaceae* |
|  | OTU109 | 0.09 | 0.87 | 96 | *Ascomycota* |  |  |  |  |  |
|  | OTU66 | 0.09 | 0.87 | 96 | *Ascomycota* | *Eurotiomycetes* | *Chaetothyriales* | *Trichomeriaceae* | *Knufia* |  |
|  | OTU233 | 0.10 | 0.85 | 93 | *Basidiomycota* | *Agaricomycetes* | |  |  |  |
|  | OTU47 | 0.10 | 0.85 | 93 | *Ascomycota* | *Sordariomycetes* | *Glomerellales* | *Plectosphaerellaceae* | *Verticillium* | *Verticillium alfalfae* |
|  | OTU22 | 0.10 | 0.85 | 93 | unclassified Fungi |  |  |  |  |  |
|  | OTU49 | 0.07 | 0.79 | 88 | *Ascomycota* |  |  |  |  |  |
|  | OTU148 | 0.07 | 0.79 | 88 | unclassified Fungi |  |  |  |  |  |
| CC4Y | OTU85 | 0.09 | 0.88 | 98 | *Ascomycota* | *Sordariomycetes* | *Hypocreales* | *Bionectriaceae* | *Clonostachys* | *Clonostachys rosea* |
|  | OTU218 | 0.09 | 0.88 | 98 | *Ascomycota* | *Dothideomycetes* | *Pleosporales* | |  |  |
|  | OTU70 | 0.09 | 0.88 | 98 | *Ascomycota* |  |  |  |  |  |
|  | OTU11 | 0.09 | 0.88 | 98 | *Ascomycota* | *Sordariomycetes* | *Hypocreales* | *Sarocladiaceae* | *Sarocladium* | |
|  | OTU181 | 0.09 | 0.88 | 98 | *Ascomycota* | *Sordariomycetes* | |  |  |  |
|  | OTU202 | 0.10 | 0.88 | 97 | *Ascomycota* |  |  |  |  |  |
|  | OTU136 | 0.10 | 0.88 | 97 | *Ascomycota* | *Sordariomycetes* | *Hypocreales* | *Bionectriaceae* | *Geosmithia* | *Geosmithia putterillii* |
|  | OTU56 | 0.10 | 0.88 | 97 | unclassified Fungi |  |  |  |  |  |
|  | OTU240 | 0.12 | 0.87 | 95 | unclassified Fungi |  |  |  |  |  |
| CC5Y | OTU118 | 0.27 | 0.96 | 104 | unclassified Fungi |  |  |  |  |  |
|  | OTU111 | 0.27 | 0.96 | 104 | *Ascomycota* |  |  |  |  |  |
|  | OTU20 | 0.20 | 0.84 | 96 | *Basidiomycota* | *Tremellomycetes* | *Cystofilobasidiales* | *Cystofilobasidiaceae* | *Cystofilobasidium* | |
|  | OTU157 | 0.20 | 0.84 | 96 | *Ascomycota* | *Sordariomycetes* | *Hypocreales* | *Hypocreaceae* | *Kiflimonium* | *Kiflimonium curvulum* |
| CC6Y | OTU66 | 0.38 | 0.81 | 100 | *Ascomycota* | *Eurotiomycetes* | *Chaetothyriales* | *Trichomeriaceae* | *Knufia* |  |
|  | OTU28 | 0.32 | 0.77 | 99 | unclassified Fungi |  |  |  |  |  |
|  | OTU256 | 0.28 | 0.75 | 95 | *Ascomycota* | *Orbiliomycetes* | *Orbiliales* |  |  | *uncultured Orbiliaceae* |
