# Supplementary figures and images for: Impacts of Continuous Cropping on Fungal Communities in the Rhizosphere Soil of Tibetan Barley
Source: Front Microbiol. 2022 Feb 4;13:755720. doi: 10.3389/fmicb.2022.755720 (PMC8854972; doi:10.3389/fmicb.2022.755720)

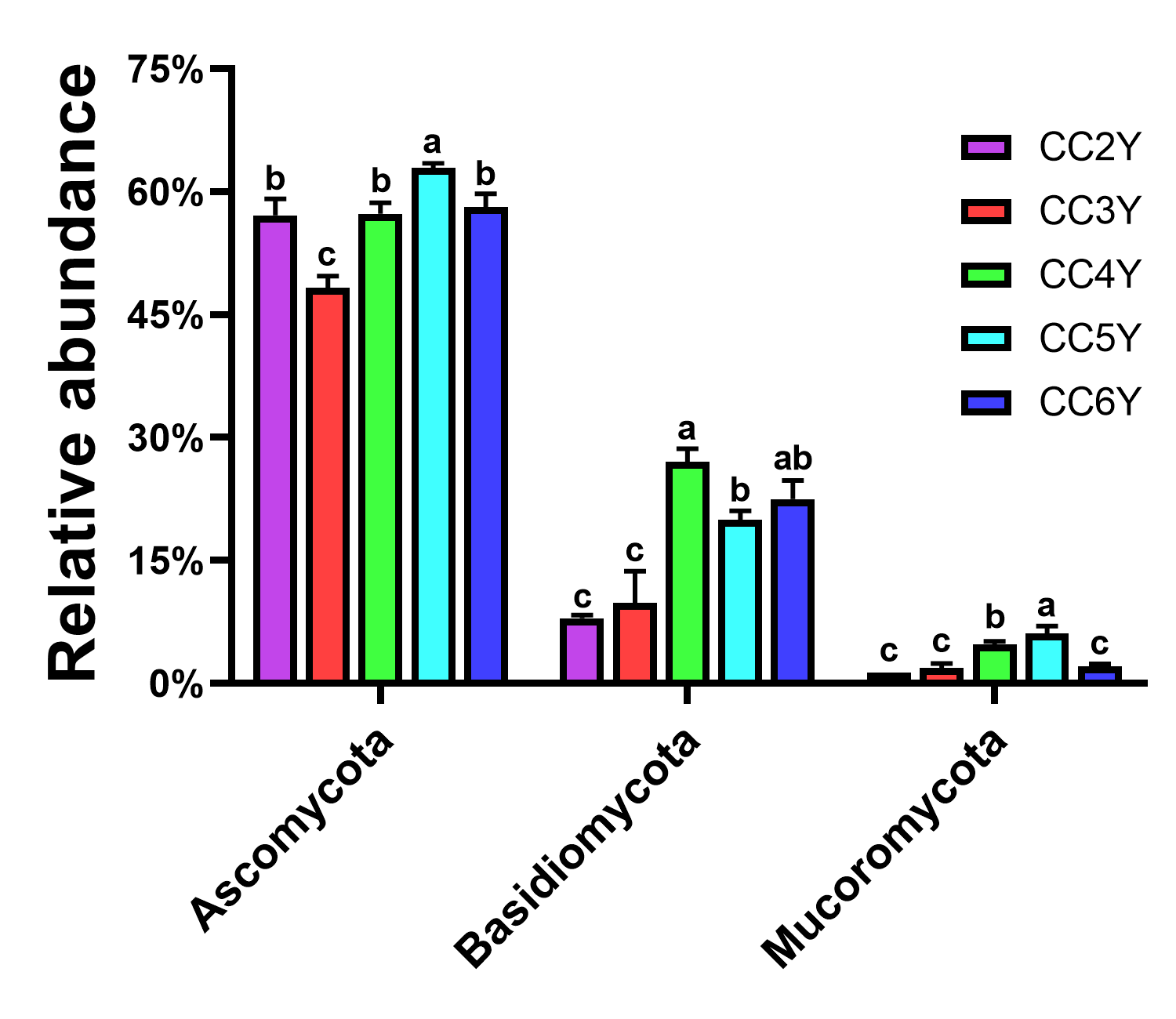

Supplement: Supplementary Figure S1 — Relative abundance of fungal phyla in the rhizosphere soil of Tibetan barley continuously cropped for different durations. Values represent means ± SD (n = 4). Different lowercase letters within the same column indicate significant differences among different continuous cropping durations at p < 0.05 according to a two-way ANOVA. [file Image_1.TIF]

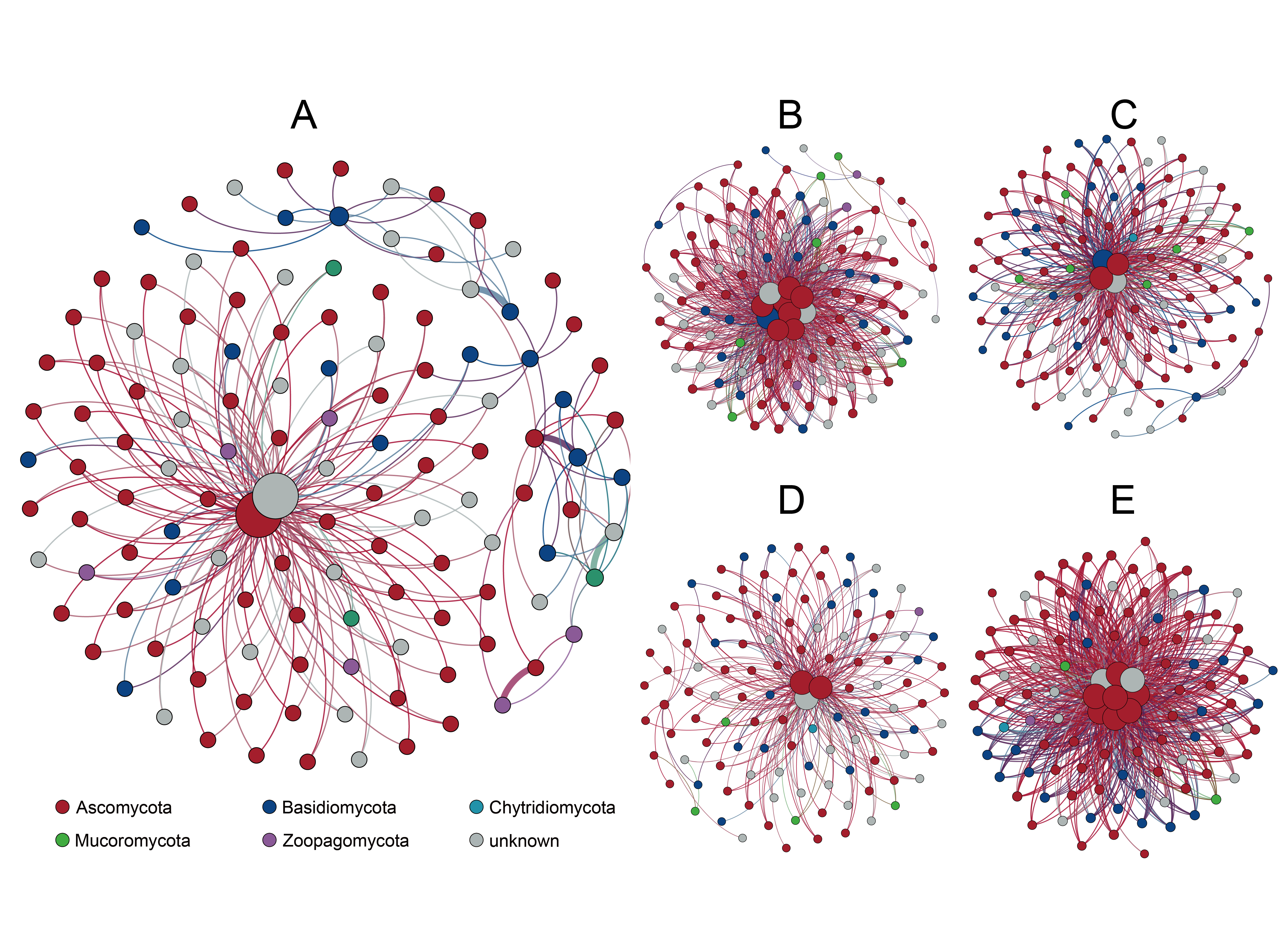

Supplement: Supplementary Figure S2 — Co-occurrence networks show the correlations among total fungal OTUs in CC2Y (A), CC3Y (B), CC4Y (C), CC5Y (D), and CC6Y (E). A connection indicates a strong (|r| of ≥ 0.8) and significant (p of ≤ 0.01) Spearman’s correlation. Red lines indicate positive correlations, while green lines indicate negative correlations. The network is colored by a module that clusters the tightly connected nodes. [file Image_2.TIF]
